# Redefining ARDS: a paradigm shift

**DOI:** 10.1186/s13054-023-04699-w

**Published:** 2023-10-31

**Authors:** Jesús Villar, Tamas Szakmany, Giacomo Grasselli, Luigi Camporota

**Affiliations:** 1 CIBER de Enfermedades Respiratorias, Instituto de Salud Carlos III, 28029 Madrid, Spain; 2 Research Unit, Hospital Universitario Dr. Negrin, Barranco de La Ballena S/N, 4Th Floor-South Wing, 35019 Las Palmas de Gran Canaria, Spain; 3 Li Ka Shing Knowledge Institute at St. Michael’s Hospital, Toronto, ON M5B 1W8 Canada; 4 Department of Intensive Care Medicine & Anesthesia, Aneurin Bevan University Health Board, Newport, NP20 2UB Wales, UK; 5 Honorary Professor in Intensive Care, Cardiff University, Cardiff, CF14 4XW Wales, UK; 6 Department of Anesthesia, Critical Care and Emergency, Fondazione IRCCS Ca’ Granda Ospedale Maggiore Policlinico, Milan, Italy; 7 Department of Pathophysiology and Transplantation, University of Milan, Milan, Italy; 8 Department of Adult Critical Care, Guy’s and St Thomas’ NHS Foundation Trust, London, UK; 9 Centre for Human and Applied Physiological Sciences, King’s College London, London, UK

**Keywords:** Acute respiratory distress syndrome, Definitions, Acute hypoxemic respiratory failure, Mechanical ventilation, Standardization, Stratification, Prognosis, Clinical trials

## Abstract

Although the defining elements of “acute respiratory distress syndrome” (ARDS) have been known for over a century, the syndrome was first described in 1967. Since then, despite several revisions of its conceptual definition, it remains a matter of debate whether ARDS is a discrete nosological entity. After almost 60 years, it is appropriate to examine how critical care has modeled this fascinating syndrome and affected patient’s outcome. Given that the diagnostic criteria of ARDS (e.g., increased pulmonary vascular permeability and diffuse alveolar damage) are difficult to ascertain in clinical practice, we believe that a step forward would be to standardize the assessment of pulmonary and extrapulmonary involvement in ARDS to ensure that each patient can receive the most appropriate and effective treatment. The selection of treatments based on arbitrary ranges of PaO_2_/FiO_2_ lacks sufficient sensitivity to individualize patient care.

## Problems with ARDS definitions

### Clinical vignette

A patient is hospitalized with worsening sepsis secondary to a urinary tract infection and develops dyspnea, hypoxemia and increased respiratory effort with radiographic evidence demonstrating new diffuse pulmonary infiltrates. The patient is transferred to the intensive care unit (ICU) where clinicians commenced on high-flow nasal oxygen (HFNO). After several hours, the work of breathing remains elevated and there is SpO_2_ 90% despite a HFNO at 50 L/min. As such, the patient is intubated and connected to mechanical ventilation (MV) with a tidal volume (VT) of 7 ml/kg predicted body weight (PBW) and a positive end-expiratory pressure (PEEP) of 12 cmH_2_O. The patient’s PaO_2_ increases to 160 mmHg with a FiO_2_ 0.5 (PaO_2_/FiO_2_ ratio 320 mmHg). Rapid improvement was noted following administration of antibiotics, fluids and light sedation. The patient was successfully extubated after fifty hours of MV and discharged from hospital a few days later.

### Case discussion

Did this patient have acute respiratory distress syndrome (ARDS)? According to the current Berlin definition [1], this patient met the criteria for moderate/severe ARDS, based on the acuity of presenting symptoms, the radiographic evidence of bilateral pulmonary infiltrates and the initial SpO_2_/FiO_2_ ratio when receiving HFNO therapy. The patient, however, no longer met diagnostic gas-exchange criteria after only a few hours of MV. Such a rapid recovery is conceptually inconsistent with the natural history of ARDS. This case serves to highlight several major issues with the current ARDS definition and its management. Firstly, the PaO_2_/FiO_2_ ratio is largely a function of ventilator settings [2]. Secondly, it is plausible that the PaO_2_/FiO_2_ ratio on MV would have been below 150 mmHg had the clinicians opted for a PEEP < 12 cmH_2_O. A ratio of this level may have prompted the clinicians to escalate the respiratory support for use of neuromuscular blocking agents to paralyze the patient or use of prone positioning. The apparent ‘need’ to utilize these techniques would likely delay the patient’s weaning and extubation while increasing their risk of iatrogenic complications. A single measurement of PaO_2_/FiO_2_ on admission, prior to any treatment optimization particularly if at relatively low PEEP, as indicated by the Berlin definition [1], has shown poor performance for predicting ARDS severity [3] (Table 1).

**Table 1:** Limitations of the current definition and diagnostic/therapeutic approach of ARDS

Bilateral and diffuse pulmonary edema	Lack of a marker of non-cardiogenic origin of pulmonary edema
	Lack of a (bio)marker of pulmonary vascular permeability
Oxygenation	A single measurement of PaO_2_/FiO_2_ at ARDS onset or diagnosis has poor performance for definition or predicting severity
	Lack of standardization of respiratory support settings for measuring PaO_2_/FiO_2_
	Difficult to distinguish ARDS from acute hypoxemic respiratory failure since clinical features and etiologic causes are similar
Lung mechanics	Not required in the current definition
	Missing dead space (VD/VT) measurement in definition and progression
	Hard to conceive a mechanically ventilated ARDS patient receiving PEEP ≤ 5 cmH_2_O
Systemic inflammation	Definition and categorization do not account for non-pulmonary organ failure, which is present in most patients and a major determinant of outcome
	Too much emphasis on the alveolar side. Little consideration for the pulmonary vascular and endothelial side, presence of pulmonary hypertension or right ventricular function
	Systemic inflammation seen in ARDS based on protein and mRNA biomarkers is not specific for ARDS, especially in septic patients
Categorization and sub-phenotyping	Missing stratification in sub-phenotypes based on VD/VT, endothelial injury, biomarker levels, or modifiable or treatable traits
	It is highly plausible that in a substantial proportion of patients in recent trials, the severity of lung injury was modest
Mechanical ventilation setting	It should be personalized based on etiology, lung physiology, imaging and morphology, and clinical and biological classes or subclasses
	In some ARDS trials, unselected patients could be enrolled missing the opportunity to test whether the experimental MV approach is beneficial due to lack of standardized assessment of severity prior to randomization and to lack of patient sub-phenotyping

ARDS, acute respiratory distress syndrome; mRNA, messenger ribonucleic acid; MV, mechanical ventilation; PEEP, positive end-expiratory pressure; VT/VT, dead space

### Background

The condition subsequently identified as ARDS has been known for over a century, but the first summary description of this heterogeneous pulmonary disorder was published in 1967 [4]. The clinical features included severe dyspnea, hypoxemia, decreased lung compliance and diffuse alveolar infiltrates on the chest X-ray, in a setting where cardiogenic pulmonary edema had been ruled out. Since this first description, the ARDS definition has been revised several times while many researchers and clinicians questioned its existence as a discrete entity [1, 5–7]. Authors of each revision [1, 6, 7] justified their selected criteria by pointing out the flaws in the previous definition and pledged that the “new” definition would be able to solve past shortcomings.

Each definition used the PaO_2_/FiO_2_ ratio as the main defining criterion for establishing the diagnosis and severity of the syndrome. While PaO_2_ is the most direct measurement of oxygenation status in ARDS, it is expressed in terms of PaO_2_/FiO_2_ ratio both in the AECC and Berlin definitions [1, 7]. There are no data linking PaO_2_ on a set FiO_2_ with a wide variety of ventilation settings and modes, to predictable structural changes in the alveolar-capillary membrane or to the extent of diffuse alveolar damage (DAD) at the time of ARDS diagnosis [8]. On the contrary, there is recent evidence showing a correlation between the severity of lung injury and outcome when the PaO_2_ is measured under standardized ventilatory settings [3]. Other factors affecting PaO_2_/FiO_2_ ratio include cardiac output, intrapulmonary shunt fraction, metabolic rate and hemoglobin concentration [9]. Therefore, if PaO_2_/FiO_2_ ratio is crucial to ARDS definition and its management, it should be argued that resting clinical decisions on a single value obtained outside a defined standard setting should be rejected [10]. A fundamental problem with the definitions based on criteria with such significant limitations is that operationalizing their application may affect the therapy that patients receive, or if they are enrolled into clinical trials [11], particularly in many hypoxemic patients who improve after 24 h of standard intensive care [3, 10].

## The pseudo-ARDS scenario

Various types of pulmonary and systemic insults can lead to a common pathophysiological response [12]. Regardless of the precise mechanism, the typical anatomopathological feature of ARDS is DAD [13]. In general, it is useful to think of the pathogenesis as the result of two different pathways: a direct insult to alveolar cells and an indirect insult to the endothelial cells by an acute systemic inflammatory response. The early exudative phase of DAD is characterized by inflammation and protein-rich edema [13], atelectasis and structural damage to the lung architecture if inflammation persists. Eventually, these changes evolve into a fibroproliferative phase with capillary thrombosis, lung fibrosis and neovascularization. Most ARDS patients die during this phase despite ventilatory and extracorporeal organ support.

Although there are no typical ARDS patients, it is likely that DAD is present in all of them, despite reports showing absence of DAD in a marked proportion of autopsies in patients fulfilling the Berlin criteria for ARDS [14]. This is a likely result of incorrect classification, as in those reports, lung biopsies were performed days or weeks after ARDS onset and/or initiation of therapy, and a lack of randomization in pathological studies makes difficult to determine the correlation between clinical and pathological findings. In addition, lung tissue samples reporting clinicopathological comparison with DAD [15], were obtained from patients ventilated with injurious MV settings with VT up to 16 ml/kg actual body weight [16] or PEEP from 0 to 5 cmH_2_O in most patients [17]. Criteria that are necessary for a definitive diagnosis of ARDS (increased pulmonary vascular permeability and DAD) are difficult to incorporate into clinical practice. Probably, a simple measure of vascular permeability at the bedside, such as extravascular lung water, is needed in future ARDS definitions for identifying ARDS, although how abnormal must pulmonary vascular permeability be before predicting the presence of DAD is not clearly known [8].

Many forms of acute hypoxemic respiratory failure mimic ARDS and do not have DAD, if one considers how prevalent are fluid overload, bilateral pleural effusions and bilateral atelectasis in ICU [18]. Patients with these features may meet the Berlin definition, but their overall outcome is usually better compared to true ARDS. Enrollment of patients with rapidly improving ARDS or pseudo-ARDS may contribute to the failure of therapeutic clinical trials [19], paving the way to studies where physiological enrichment is used to overcome this issue [2]. Severe hypoxemia caused by lobar consolidation is frequently treated as ARDS, when it is possible that specific treatment options would benefit these patients, while they could be spared from the development of ventilator-induced lung injury (VILI) in the unaffected lung [20].

## Problems with hypoxemia

An integral part of the supportive therapy for ARDS is the application of respiratory support aimed at achieving adequate gas-exchange and tissue oxygenation without further damaging the lungs [20]. The use of MV is vital for most ARDS patients, but over the last decade, ARDS patients with mild or moderate forms of lung injury have successfully been managed without endotracheal intubation [11], as recognized by the Berlin definition [1] and by recent guidelines [11].

We suspect that PaO_2_/FiO_2_ ratio will be not eliminated from future definitions of ARDS. Of note, a standardized level of FiO_2_ and PEEP has never been a condition for defining hypoxemia under MV. In patients fulfilling ARDS criteria, assessment at 24 h on PEEP ≥ 10 cmH_2_O with FiO_2_ ≥ 0.5 for 30 min caused PaO_2_/FiO_2_ ratio to increase, such that more than a third of patients no longer met ARDS criteria [3]. In addition, the exact FiO_2_ is difficult, if not impossible, to be determined in patients on non-invasive ventilation or HFNO. We suspect that none of proposed indices of oxygenation for ARDS categorization and prediction of outcome will be useful to make clinical decisions unless assessed or calculated using standardized ventilatory settings [21, 22]. In the latest iteration of the definition, some authors have proposed the use of SpO_2_/FiO_2_ ratio, mainly keeping in mind the resource constrained environments, where arterial blood gas analysis might be difficult or impossible to achieve [11]. Unfortunately, SpO_2_ is affected by several variables [23] such as changes in temperature, pH, PaCO_2_, concentration of 2,3-diphosphoglycerate and carboxyhemoglobin, and its measurement is influenced by ethnicity [24], although none of these variables affect PaO_2_. SpO_2_/FiO_2_ ratio contains all the problems of the PaO_2_/FiO_2_ ratio, with the added problem that the 95% confidence interval for SpO_2_ vs. SaO_2_ is ± 5% when patient is desaturated, and PaO_2_ values could fluctuate > 300 mmHg when SpO_2_ is ≥ 97%.

In the European Collaborative Study [25], the mortality of patients with PaO_2_/FiO_2_ < 150 mmHg at 24 h was almost double the mortality of patients with PaO_2_/FiO_2_ ≥ 150 mmHg. Three recent clinical trials used a value of PaO_2_/FiO_2_ < 150 mmHg at PEEP ≥ 5 [26, 27] or ≥ 8 cmH_2_O [28] to enroll patients during the first 24–48 h of ARDS diagnosis. It is plausible that in a substantial proportion of patients in recent clinical trials, the severity of lung injury was modest. If patients have a low risk of the condition to be prevented, any trial will not validate the value of the intervention under study [29]. In a recent study with 1303 moderate/severe ARDS patients [2], almost half of them had a PaO_2_/FiO_2_ ≥ 150 mmHg at 24 h and their ICU mortality was about 20%, whereas patients with PaO_2_/FiO_2_ < 150 mmHg had an ICU mortality greater than 45%. It is possible that in the new updated ARDS categorization, a new PaO_2_/FiO_2_ threshold could be incorporated (Table 2).

**Table 2:** Potential recommendations for improving the definition of ARDS

New datasets	1. Expiration date for observational studies and trials conducted before year 2010
Actionable criteria	2. Definition should be based on actionable and modifiable criteria, including VD/VT, lung imaging, biomarker levels, etc.
PaO_2_/FiO_2_	3. It should be assessed under standardized conditions (e.g., measured at predefined FiO_2_ and PEEP levels)
	4. Categorization may include the threshold of 150 mmHg (< 150, ≥ 150)
Measures of severity	Two measures of “true” severity of ARDS:
	5. Lung injury per se: “Severe” ARDS should not be based only on PaO_2_/FiO_2_
	6. Severity of patient illness, including comorbidities and frailty
Enrichment strategies	7. Prediction or prognostic enrichment strategies for inclusion of patients into therapeutic clinical trials. The use of artificial intelligence techniques may help
Pulmonary circulation	8. More precise information about the anatomic/physiologic state of the pulmonary vascular circulation
Stratification, classification, or sub-phenotyping	9. An updated definition requires a new categorization or classification of severity based on gas-exchange, lung imaging, VD/VT, biomarker levels, use of non-invasive mechanical ventilation, degree of vascular permeability
Broadening definition	10. Excessive broadening of criteria required to diagnose ARDS should be avoided
International professional societies	11. Recommendations for management and treatment in the new updated ARDS definition should be implemented by International Professional Societies
Implementation	12. Implementation of a “Surviving ARDS (including patients at risk for) Campaign” with frequent updates

ARDS, acute respiratory distress syndrome; VD/VT, dead space

## Future directions

**Fig. 1:**
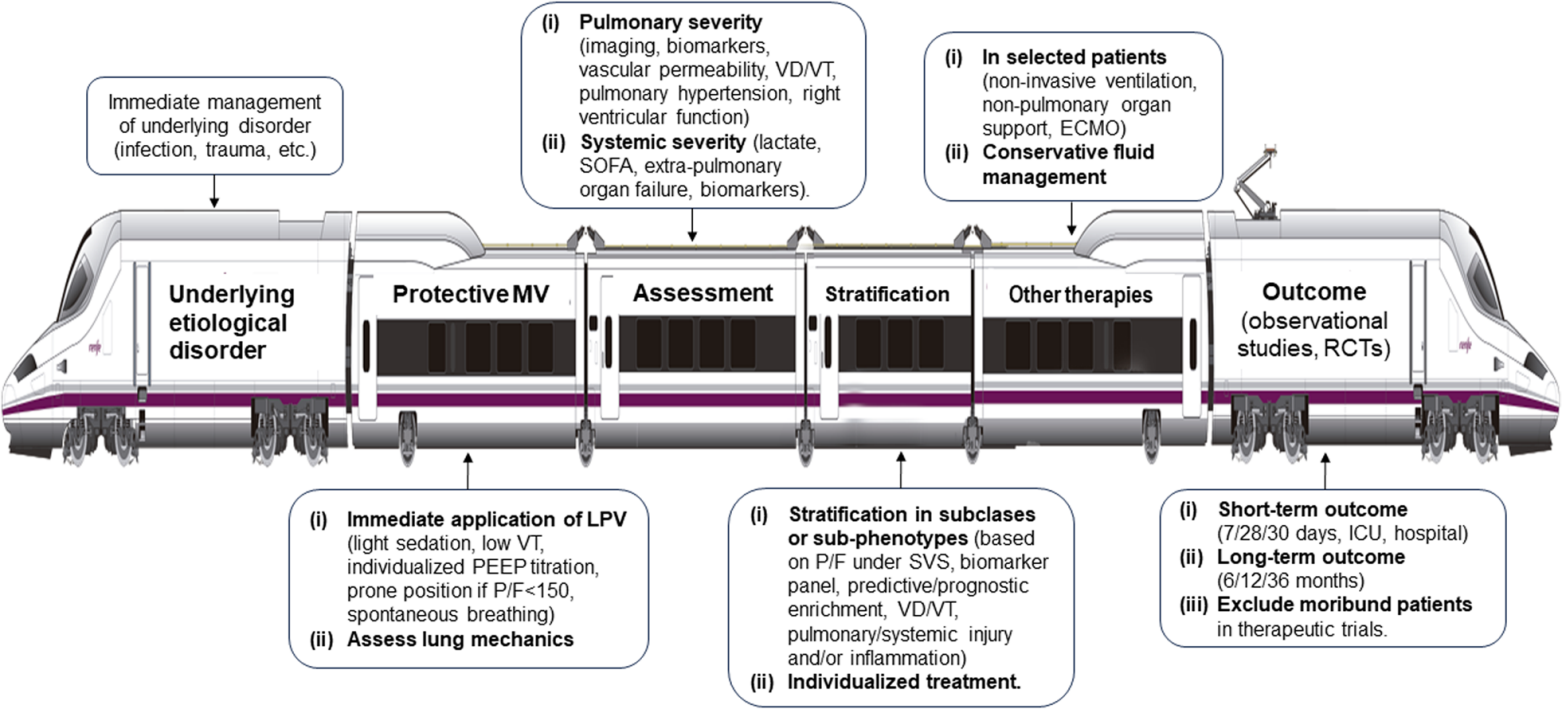
The acute respiratory distress syndrome (ARDS) high-speed train showing variables and factors affecting definition and outcome of patients with ARDS. Abbreviations: ECMO, extracorporeal membrane oxygenation; ICU, intensive care unit; LPV, lung protective ventilation; MV, mechanical ventilation; PEEP, positive end-expiratory pressure; P/F, PaO_2_/FiO_2_ ratio; RCT, randomized controlled trials; SOFA, sequential organ function assessment; SVS, standardized ventilator settings; VD/VT, alveolar dead space; VT, tidal volume

We believe that the term ARDS should be used with greater care. As suggested by experts in the field of critical illness, we believe that the current ARDS-based framework of illness should be reconsidered [30]. Clinicians should be interested in operational definition criteria that can trigger the use of therapies with high probability of resulting in improved outcomes (Table 2). To quantify accurately the severity of ARDS, we would ideally need two indices of severity: one that measures the severity of lung injury per se, and another that measures the overall severity of patient’s overall illness which would then quantify the context within which ARDS develops [8, 31]. Without those measures and understanding the effect of specific etiologies on the outcome (Fig. 1), any new updated definition of ARDS will be a perpetual iteration of the same shortcoming without a substantial advancement since its first description [32]. Subdividing ARDS patients into categories reflecting different severities or modifiable pathophysiological processes represents the most critical advance for precision medicine in ARDS. It provides a rationale for identifying patients that are resistant to therapy, or who should be the target for aggressive and innovative therapies, or in whom endotracheal intubation and MV could be avoided, or who should be excluded from some clinical trials [33–35]. Most studies on sub-phenotypes in ARDS to date are based on retrospective analyses [36] and it is unclear whether those subtypes of patients represent categorization of the etiologic underlying disease or of ARDS itself [30, 37]. Even with this caveat, it is possible to combine information obtained from lung imaging and pulmonary/systemic biomarkers to personalize individual management of ARDS [38].

ARDS is frequently associated with hemodynamic instability, one of the main determinants of mortality. There is a place for invasive hemodynamic monitoring in patients who need an accurate assessment of their cardiovascular status, although the specific monitoring should be individualized. Vascular alterations in ARDS include vasoconstriction and vasodilation of pulmonary vessels leading to unfavorable blood flow distribution, pulmonary hypertension and right ventricular dysfunction [39, 40]. Management of intravenous fluids and vasopressors in ARDS is a key challenge and a top research priority. One should consider the risks and benefits in each phase of ARDS and facilitate fluid removal. As reported in a recent study, clinicians administer higher doses of fluids and lower doses of vasopressors than recommended by a machine learning (ML) model [41]. Of note, patients receiving doses similar to those recommended by the ML model had the lowest mortality rate.

Greater emphasis should be placed on the role of carbon dioxide (CO_2_) and dead space (VD/VT) in determining the severity of disease [42]. VD/VT or wasted ventilation (the portion of VT that does not participate in gas-exchange) is not included in any definition of ARDS (Table 1). Elevated VD/VT is associated with lower probability of being discharged alive [43, 44]. The lack of precise information about the anatomic state of the pulmonary vascular circulation makes difficult to establish a rational criterion for ARDS stratification and for initiating specific therapy. Analysis of expired CO_2_ kinetics provides important non-invasive cardiorespiratory information for clinical assessment, monitoring and management of ventilated ARDS patients. The concept of VD/VT is clinically useful not only to assess and adjust alveolar ventilation during MV but also to detect alveolar overdistension [42].

We do not know yet whether favoring early spontaneous ventilation in ARDS improves outcome when compared to controlled MV plus sedation and proning [45, 46]. In managing ARDS, the underlying disorders lead to a high respiratory drive and should be addressed immediately following intubation. Allowing early spontaneous breathing as soon as some improvements occur could decrease duration of MV. Early spontaneous breathing could allow to use high levels of PEEP to prevent atelectrauma and inflammation for enhancing the lung to heal [46].

Finally, future research should address precision medicine in ARDS, invoking the concept of treatable traits [30]. We need clinical trials comparing current management with that derived from precision medicine. No tools currently exist to personalize treatment of ARDS and assist clinicians in making decisions in real time at the bedside. Features of a ML model to predict ICU mortality suggested that they were clinically interpretable and relied primarily on sensible clinical and biological parameters [31].

## Data Availability

When writing the manuscript, the authors did not have access to any special sets of data. As such, the authors cannot provide any special access to datasets that readers might request.
